# Boston Society for Medical Improvement. Salutary Influences of Medical Associations

**Published:** 1846-07

**Authors:** 


### EDITORIAL DEPARTMENT.

*Boston Society for Medical Improvement. Salutary influences of Medical Associations.*—During a recent visit at Boston, Mass., we had the pleasure of attending a meeting of the Society for Medical Improvement. This is an association of the Physicians of that city, embracing all in regular standing who choose to avail themselves of its advantages. The meetings are held bi-weekly, in rooms exclusively devoted to purposes of the Society. At their regular meetings, a stated essay, or report on some medical subject, is read by one of the members agreeably to appointment, and voluntary remarks and communications are offered, in turn, by any, or all present. On the evening of our attendance, a highly interesting and valuable paper on the subject of vital statistics was read by O. W. Holmes, M. D.; and we observed that every member present, when called upon in succession by the chairman, had something to offer relating to their experience, observations, or reading since the preceding meeting of the Society. Whenever a subject was introduced by a member, all present who chose, expressed their views in a colloquial, informal manner, and after it had received all the attention or discussion they were disposed to give to it, the chairman called upon another member for a fresh topic. In this manner, during the evening, a variety of interesting and useful matter was introduced.

The rooms occupied by the Society are comfortably furnished, and contain a valuable pathological cabinet — the property of the Society. This cabinet consists of a variety of dried and wet preparations, casts, drawings, &c., illustrating all classes of pathological lesions. Many of the specimens are very rare and valuable. Dr. J. B. S. Jackson, one of the most distinguished pathological anatomists of our country, has especially contributed toward the formation of this cabinet. Through his polite attentions we enjoyed the gratification of examining the collection in detail, and of availing ourselves of his intimate knowledge of the circumstances connected with their individual history, as well as of the subject in general.

Societies of this kind are of very great utility. In the first place, they conduce to mutual improvement, by the interchange of views, the collection of facts, and the motives felt by every member for personal observation and reflection, in order that each may contribute his quota of useful information for the benefit of the whole. But, in the second place, what is even more important, they serve to unite the profession as members of a fraternity, to soften asperities occasioned by mutual jealousy and distrust, obviate dissension, and secure harmony and good feeling. These results are of inestimable importance. No man of generous feelings, or right sentiments, can, or ought to feel satisfied to practice his profession, without the respect and confidence of his brethren in the profession, not less than the approbation of the community in which he dwells. No honorable, high minded man, assuredly, would covet opposition and distrust from his fellow-practitioners. But many are obliged to submit to an absence of all friendly relations with those engaged in the same calling, simply because they do not know each other except through the medium of suspicions, often entirely unwarrantable, or the misrepresentations of false friends, who do what they can to promote mutual envy and uncharitableness. Under these circumstances, frequent, cordial intercourse is all that is necessary to correct the evil. Let the physicians of each place associate together, and commune respecting the ends and means of a common, humane, noble pursuit, and they will find it difficult to entertain toward each other feelings of animosity, and still more difficult to allow such feelings to actuate their conduct. Not only are individual comfort and respect in this way preserved, but the character of the profession with the public elevated. We have no hesitation in declaring that in our opinion, to the external relations of medical men toward each other, more than any other single cause, is due the fact that in the public mind the medical profession, as a profession, is so little esteemed. Individual practitioners may acquire consideration as practitioners, but they derive little or nothing from the profession. The latter is but respectable ; it confers no honor or dignity in itself, and the reason of this, we repeat, is, in a great measure, because the members of the profession have not themselves manifested proper respect for their profession. What they have claimed and sought for, has been their own individual success and preferment, without reference to the whole profession ; and, indeed, too often at the expense of their fellow practitioners. The character of the profession has been with them of minor importance, or of none whatever. Hence, while they have been willing to appropriate whatever reputation they have been able to acquire as individuals, they have concurred with the disposition around them to depreciate other members of the profession, forgetting that public estimation of the profession is based upon respect for its members in the aggregate, not in particular instances, which are liable to be set down as exceptions to a general rule. Of course, in these remarks we do not intend to make a sweeping assertion, applicable to the whole medical faculty. We refer only to a spirit which is too common in the ranks of the profession, as all who have had their thoughts directed to the subject must admit ; and as a remedy for which we would have in every city and village a Medical Society, instituted and conducted upon right principles ; and in furtherance of the same object, we desire to see a National Association instituted by the medical profession of this country "for the protection of their interests, maintenance of their honor and respectability, for the advancement of their knowledge, and the extension of their usefulness."*

* Quoted from the first of the resolutions adopted by the late National Medical Convention.

